# microRNA-150 promotes cervical cancer cell growth and survival by targeting FOXO4

**DOI:** 10.1186/s12867-015-0052-6

**Published:** 2015-12-29

**Authors:** Jun Li, Lina Hu, Chao Tian, Feng Lu, Jia Wu, Li Liu

**Affiliations:** Department of Obstetrics and Gynecology, The Second Affiliated Hospital, Chongqing Medical University, Linjiang Road 76, Chongqing, 400010 People’s Republic of China; Department of Obstetrics and Gynecology, The Second Clinical Medical Institute of North Sichuan Medical College, Nanchong, 637000 Sichuan People’s Republic of China

**Keywords:** Cervical cancer, miR-150, FOXO4, Apoptosis, Cell cycle

## Abstract

**Background:**

Dysregulation of microRNA-150 (miR-150) is commonly observed in solid tumor and has been reported to be involved in multiple important biological processes, such as cell proliferation, apoptosis, and metastasis. Elevated miR-150 level was also detected in cervical carcinoma, whereas its function in cancer progression has not been studied yet.

**Methods:**

The expression of miRNA-150 in cervical carcinoma was compared with normal cervical tissue and using qRT-PCR. The effects of miR-150 on cell cycle and apoptosis, as well as the expression of cycle- and apoptosis-related genes, were determined using flow cytometry, TUNEL assay, qRT-PCR, and Western blot, respectively. The direct target of miR-150 was confirmed using 3′ untranslated region (UTR) luciferase reporter assay.

**Results:**

miR-150 promotes cervical cancer cell survival and growth, while the inhibition of miR-150 suppresses these actions. miR-150 also induced the cell cycle progression from G1/G0 to S phase, resulting in an enhancement of growth. Several cell cycle- and apoptosis-related genes, CyclinD1, p27, BIM, and FASL were modulated by miR-150. Moreover, miR-150 directly reduced the expression of FOXO4, which regulates the expression of CyclinD1, p27, BIM, and FASL, by targeting its 3′ UTR.

**Conclusion:**

Taken together, our data demonstrated that elevated miR-150 targets FOXO4 expression and therefore regulates multiple genes expression, resulting in cervical cancer cell growth and survival.

## Background

Uterine cervical cancer is the third most common cancer, and is one of the leading causes of cancer mortality in young women worldwide [1–3]. Over 500,000 new cases and 274,000 death in global are reported each year [3]. Several treatment strategies, surgery, chemotherapy, radiotherapy and combined radio-chemotherapy were used in the clinic depending on the stage of cancer, and overall survival rate was improved. However, treatment failure and poor outcome occurs, especially in advanced stage of cervical cancer, due to the development of resistance to radiotherapy and chemotherapy [4, 5]. Thus, identification of new potential therapeutic targets in cervical cancer is necessary for improving the treatment and prognosis.

MicroRNAs (miRNAs), endogenous non-coding RNA molecules, modulate multiple important biological processes, including cell development, proliferation, differentiation, apoptosis, signal transduction, and tumorigenesis, via regulation of genes expression [6, 7]. Dysregulation of miRNA expression is commonly observed in human malignancies and has been reported to promote the cancer progression. Aberrant expression of oncogenic and tumor suppressive miRNAs was detected in cervical cancer cell lines and they are required for tumor cell growth [8]. A study has identified 68 up-regulated miRNAs including miR-150 in the invasive cervical squamous cell carcinomas as compared with normal samples [9]. Upregulated miR150 expression was also found in cervical intraepithelial neoplasia, a well-defined precursor stages of squamous cell carcinomas [10]. Indeed, higher expression of miR-150 was found in many solid cancer types, such as gastric cancer, breast cancer, and non-small cell lung cancer, as well as cervical cancer [11–13]. miR-150 promotes the proliferation of gastric cancer and lung cancer cells through negative regulation of the pro-apoptotic gene early growth response factor 2 (EGR2) and by targeting p53, respectively, suggesting its pro-tumorigenic function [12, 14]. Multiple targets of miR-150 including mucins 4 (MUC4), zinc‑finger E‑box binding homeobox 1 (ZEB1), EGR2, p53, P2X purinoceptor 7 (P2X7), SRC kinase signaling inhibitor 1 (SRCIN1), BRI1‑associated receptor kinase 1 (BAK1), and C-Myb, were also identified in several cancer cells and these targets are involved in the cell proliferation, apoptosis, and metastasis [12, 13, 15–18]. However, the functions of miR-150 in the development of cervical cancer remain unclear.

In the present study, we demonstrate the elevated expression of miR-150 in cervical carcinoma as compared with para-carcinoma tissues and normal tissue from healthy donors, and that increased miR-150 expression is associated with the processed stages of cancer. Our data indicate that miR-150 targets FOXO4 and regulates multiple proliferation- and apoptosis-related proteins, therefore, promoting cervical cell survival and growth.

## Methods

### Patients and cell culture

Carcinoma and para-carcinoma tissues samples were collected from patients who are newly diagnosed with cervical carcinoma and normal cervical tissue was obtained from healthy donors. Patients and healthy donors were recruited from the second affiliated hospital of Chongqing Medical University and the second clinical medical institute of North Sichuan Medical College between July 2012 and December 2014. Verbal informed consent was obtained from all the patients and donors. The design and implementation of this research was approved and documented by the clinical research ethical committee of Chongqing Medical University in Chongqing and North Sichuan Medical College in Nanchong. The human cervical carcinoma cell line C-33A and human embryonic kidney cell line 293T was used in this study and maintained respectively in MEM and DMEM medium supplemented with 10 % heat-inactivated fetal bovine serum, glutamine, and antibiotics at 37 °C in 5 % CO_2_.

### miRNA/RNA isolation and real-time PCR

miRNA was isolated from tissues or cells using miRcute miRNA Kit (Tiangen, China) according to the manufacturer’s instructions. MiScript II RT kit (Qiagen, Germany) was used for reverse transcription of miRNA. The expression of miR-150 was determined by miScript SYBR Green PCR Kit (Qiagen), using real-time PCR. The primers for miR-150 and U6 were purchased from Qiagen. Ct value of miR-150 was normalized to the U6 and the relative expression was carried out by 2^−ΔΔCt^ method. Total RNA was isolated from C-33A cells using RNAprep pure Micro Kit (Tiangen) and reverse transcription of mRNA was performed using QuantScript RT kit (Tiangen) according to the manufacturer’s protocol. RT-PCR was performed with miScript SYBR Green PCR Kit (Qiagen) and specific primers using the ABI 7900TH Real-Time PCR System (Applied Biosystems, USA). The specific primer sequences for the genes were shown in Table 1. Relative mRNA expression was carried out using 2^−ΔΔCt^ method after normalization to GAPDH.

**Table 1:** Primer sequences used for RT-PCR

Gene	Primer (forward) 5′–3′	Primer (reverse) 5′–3
GAPDH	CGCTCTCTGCTCCTCCTGTT	CCATGGTGTCTGAGCGATGT
CyclinD1	CAATGACCCCGCACGATTTC	CATGGAGGGCGGATTGGAA
p27	AACGTGCGAGTGTCTAACGG	CCCTCTAGGGGTTTGTGATTCT
FASL	CTCCGAGAGTCTACCAGCCA	TGGACTTGCCTGTTAAATGGG
BIM	TAAGTTCTGAGTGTGACCGAGA	GCTCTGTCTGTAGGGAGGTAGG

### Transient transfection

For transient transfection, miR-150 mimics (50 nM, Qiagen), miR-150 inhibitors (50 nM, Qiagen), or a negative control siRNA were used and transfected into C-33A cells using the Lipofectamine 2000 Transfection Regent (Invitrogen, USA) according to the manufacturer’s instructions.

### Establishment of cell lines expressing miR-150 mimics or inhibitors

Lentiviral vectors were used to establish stable cell lines expressing miR-150 mimics or inhibitors. Psin-EF2-miR-150-IRES-GFP-puro expressing miR-150 pre-miRNA was constructed using specific primers, 5′-GCCGAATTCGCATAGGGTGGAGTGGGTGT-3′ (forward) and 5′-GCCGGATCCATAGAAACAGGTGTACTTTG-3′ (reverse). The paired miR-150 inhibitors (forward: 5′-GATCCCCCACTGGTACAAGGGTTGGGAGA-3′, reverse: 5′-AATTCTAAAAATCTCCCAACCCTTGTACCAGTG-3′) were inserted into pshRNA-copGFP Lentivectors. 293T cells were used to generate lentivirus containing the sequences of miR-150 precursor or inhibitors. 293T cells were co-transfected with pMD2.G, pSPAX2, and pshRNA-copGFP-inhibitor or Psin-EF2-miR-150-IRES-GFP-puro using Lipofectamine 2000 Transfection Regent (Invitrogen). After 48 h, supernatant was collected and filtered using 0.22 um filter. Filtered supernatant containing lentivirus was next used for transduction. C-33A cells were infected with lentivirus for 24 h and cultured with MEM medium including puromycin (5 ng/mL). Expand cells were then used for further experiments and transduction efficiency was measured by a fluorescence microscope (Olympus, Japan). A control cell line transducted with empty vectors was also established after the same procedure.

### Terminal deoxynucleotidyl transferase dUTP nick end labeling (TUNEL) assay

TUNEL assay with propidium iodide (PI) staining was used to identify apoptosis.C-33A cells transfected with miR150 mimics or inhibitors for 48 h were fixed and staining with fluorescein-conjugated dUTP (green) and PI (red). The apoptotic or living cells were observed by a fluorescence microscope (Olympus, Japan). This assay was independently repeated for 3 times.

### Cell growth assay

C-33A cells (1 × 10^4^) expressing miR-150 mimics or inhibitors, as well as control cells, were cultured in a 24-well plate for 4 days, and the cell number of the cells was determined every day. This assay was performed in triplicate and repeated in 3 independent experiments.

### Cell cycle analysis

Cells were collected and washed with phosphate-buffered saline (PBS), fixed in cold 70 % ethanol at 4 °C overnight. Fixed cells were washed with PBS and incubated with RNase A (100 µg/ml) and PI (50 µg/ml) for 30 min. The DNA content was determined using a FACSCanto flow cytometer (BD Biosciences, USA) and the cell cycle profile was evaluated by FACS Diva Software (BD Biosciences). This assay was repeated in 3 independent experiments.

### Western blot

Whole cell lysates were extracted from cells suspended in radio immune precipitation buffer (Beyotime, China). Lysates were separated by electrophoresis on polyacrylamide gels containing 0.1 % sodium dodecyl sulfate (SDS-PAGE) and then transferred to polyvinylidene difluoride membranes. The membranes were blocked with 5 % non-fat milk in Tris Buffer Saline Tween 20 (TBST) buffer. The blots were incubated with the primary antibody and exposed to HRP-conjugated secondary antibody. The bands on the membrane were visualized and captured using the ECL reagent (Beyotime) and X-ray films, respectively. The pixel densities of proteins were quantified using ImageJ 1.47 software. Antibodies against to GAPDH, CyclinD1, p27, p-pRb, FasL, and Bim, as well as horseradish peroxidase (HRP)-linked anti-mouse and -rabbit IgG, were bought from Beyotime and used in this study.

### Luciferase reporter assay

Wild type or mutant 3′ untranslated region (UTR) of FOXO4 containing the putative binding sites for miR-150 were subcloned into psiCHECK-2 Vectors. The constructed plasmids were transfected into C-33A cells or cells expressing miR-150 mimics or inhibitors using Lipofectamine 2000 Transfection Regent (Invitrogen). After 48 h, the luciferase activity in cell lysates was evaluated using the Dual-Luciferase Reporter Assay system according to the manufacturer’s instructions (Promega, USA).

### Statistical analysis

Statistical significance for the experiments was determined using the Student’s *t* test or One-way ANOVA. p <0.05 was regarded as statistically significant. The standard deviation was demonstrated by bar in the figures.

## Results

### Elevated expression of miR-150 in cervical cancer

**Fig. 1:**
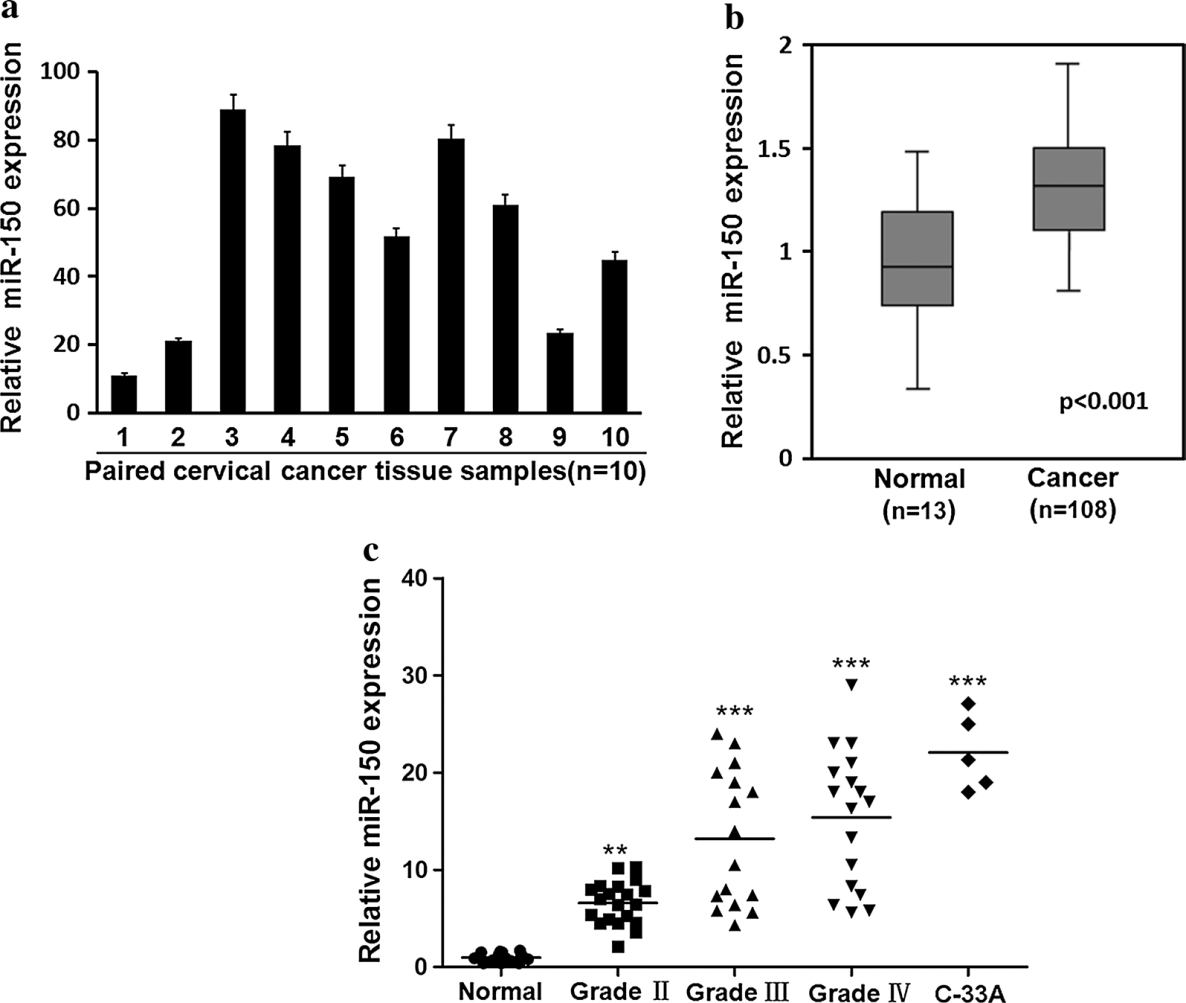
Cervical cancer cells express higher level of miR-150. **a** miR-150 expression was measured in cervical carcinoma and para-carcinoma tissues from the patients (n = 10) by RT-PCR. The miR-150 expression in cervical carcinoma tissue normalized to that in paired para-carcinoma tissues is presented by a histogram. **b** miR-150 expression in cervical tissue from healthy donors (*normal*, n = 13) or in cervical carcinoma tissue from patients (n = 108) was determined by RT-PCR and the relative miR-150 expression is presented after normalization to healthy donors. **c** The miR-150 expression in cervical tissue from healthy donors (n = 13) or in cervical carcinoma tissue from 62 patients at different stages (23 at *grade II*, 19 at *grade III*, 20 at *grade IV*), as well as in a human cervical carcinoma cell line C-33A, was evaluated by RT-PCR

miR-150 dysregulation has been found in several solid tumors including gastric cancer, breast cancer, and lung cancer [13], whereas the relationship between miR-150 expression and cervical cancer has not been well studied. Here, we first compared the miR-150 expression in cervical carcinoma and para-carcinoma tissues obtained from 10 patients and significantly higher expression of miR-150 was observed in carcinoma cells (Fig. 1a). Moreover, the expression of miR-150 in the cervical carcinoma of cancer patients was significantly higher than that in normal cervical tissue from healthy donors (Fig. 1b). The level of miR-150 expression in cervical tissue was increased along with the stage progression and a 25 times higher miR-150 expression was found in the advanced stage of cervical cancer (Fig. 1c). A human cervical carcinoma cell line C-33A also expressed a high level of miR-150 (Fig. 1c) as compared with normal donors. These results suggest a close relationship between miR-150 expression and cervical carcinoma.

### miR-150 promotes the survival of cervical cancer cells

**Fig. 2:**
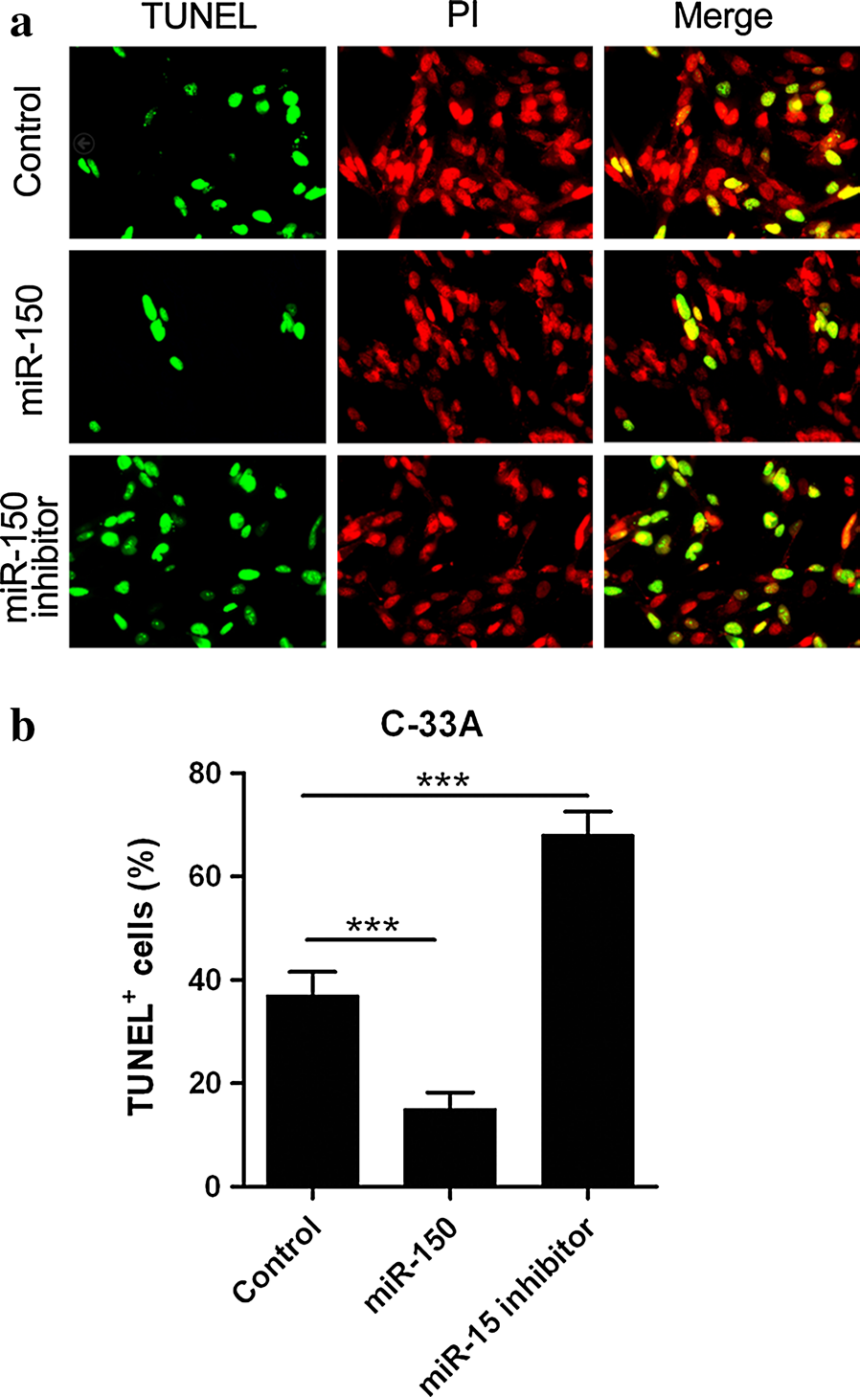
miR-150 promotes the survival of cervical carcinoma cells. **a** C-33A cells were transfected with siRNA control, or miR-150 mimics (miR-150) or inhibitors for 48 h and the apoptosis was determined by TUNEL assay and PI staining. Representative pictures of the TUNEL assays are presented. **b** The percentage of TUNEL-positive cells was counted and presented by a histogram. Mean values ± standard deviation (SD) for 3 independent experiments is shown

miR-150 has been reported to be involved in cancer cell growth and apoptosis and we next determined the functions of miR-150 in cervical carcinoma cells. C-33A cells were transfected with siRNA control, miR-150 mimics or inhibitors and cultured in serum free medium for 48 h. Thereafter, the apoptosis in these cells were determined by TUNEL assay. As shown in Fig. 2a, b, clearly decrease of apoptosis (green) was observed in the cells transfected with miR-150 mimics, whereas the miR-150 inhibitors induced more apoptotic cells (TUNEL^+^ cells), indicating the positive function of miR-150 in cervical carcinoma cell survival.

### miR-150 facilitates cervical cancer cell growth

**Fig. 3:**
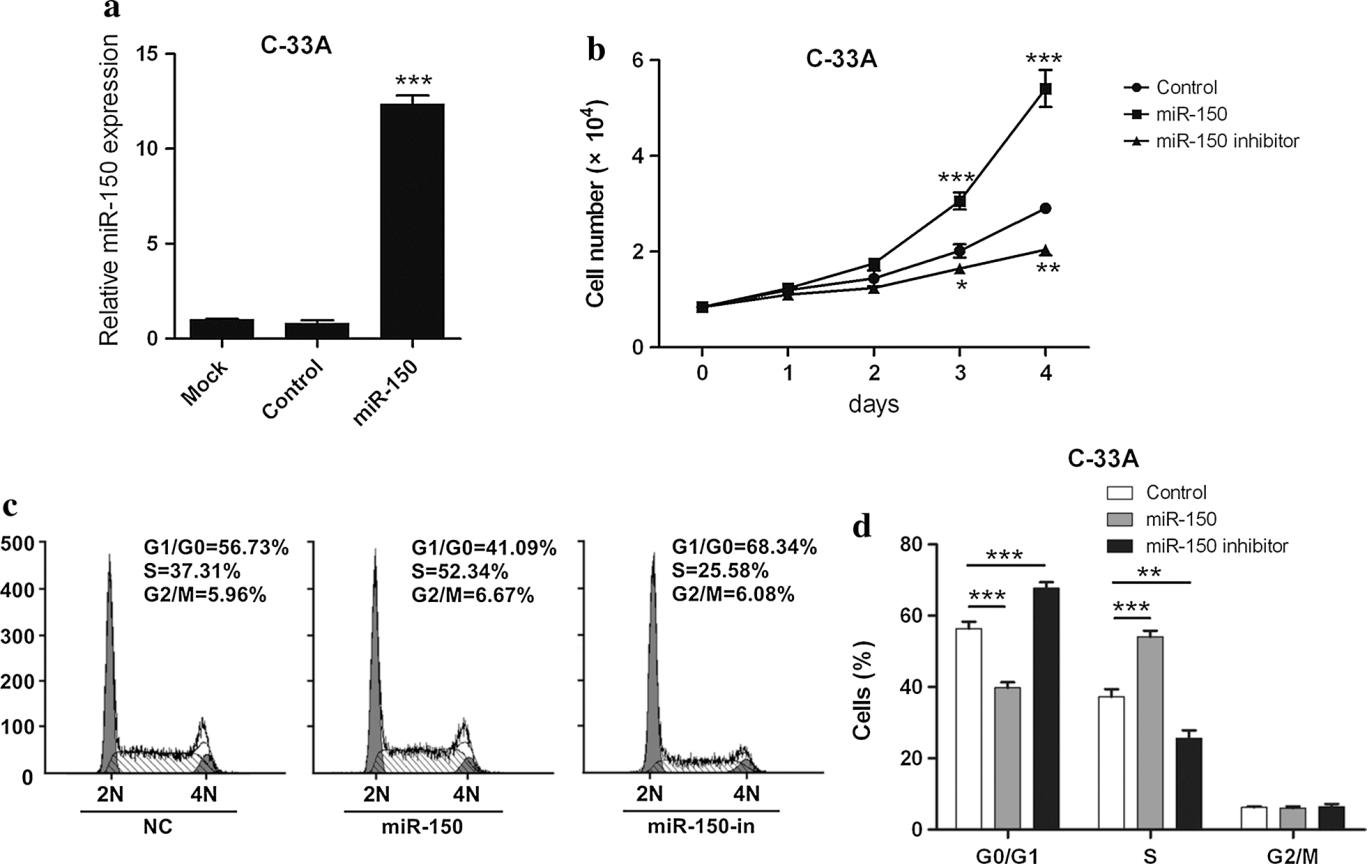
miR-150 promotes the growth of cervical carcinoma cells. **a** The expression of miR-150 in non-transduction C-33A cells (*mock*) or stable cells after transduction with control virus (*control*) or virus containing miR-150 mimics (*miR-150*) was analyzed by RT-PCR. The *bar* represents 3 independent experiments. **b** Same number of C-33A cells expressing miR-150 mimics or inhibitors, or empty vector-transducted C-33A cells (*control*) were seeded into 24-well plate and cultured for 4 days. The cell number was counted every day. Mean values ± SD for 3 independent experiments is shown. **c** The cell cycle analysis in the three stable C-33A sub-cell lines was performed after PI staining and representative DNA content profiles of cell cycle are presented. **d** The percentages of cells in different cell cycle stages from 3 independent experiments were analyzed and presented by a histogram

To further investigate the roles of miR-150 in cervical carcinoma cells, two sub-cell lines of C-33A consistently expressing miR-150 mimics or inhibitors and a control cell line were established. Overexpression of miR-150 in the cell lines expressing miR-150 mimics was confirmed by RT-PCR (Fig. 3a). The growth of these three cell lines were determined next and the cells expressing miR-150 mimics were growing faster than the control and the cells expressing miR-150 inhibitors (Fig. 3b), whereas the cells expressing miR-150 inhibitors were growing slower than the control (Fig. 3b). Cell cycle of these three cell lines was evaluated next and higher level of cell at S phase was observed in miR-150 mimic-expressing cells (Fig. 3c, d). Overexpression of miR-150 inhibitors induces more cell cycle arrest at the G1/G0 phase (Fig. 3c, d). These findings indicate that miR-150 promotes the cervical cancer cell growth and cell cycle progression from the G1/G0 to S phase.

### miR-150 regulates the expression of proteins related to cell proliferation and apoptosis in cervical cancer cells

**Fig. 4:**
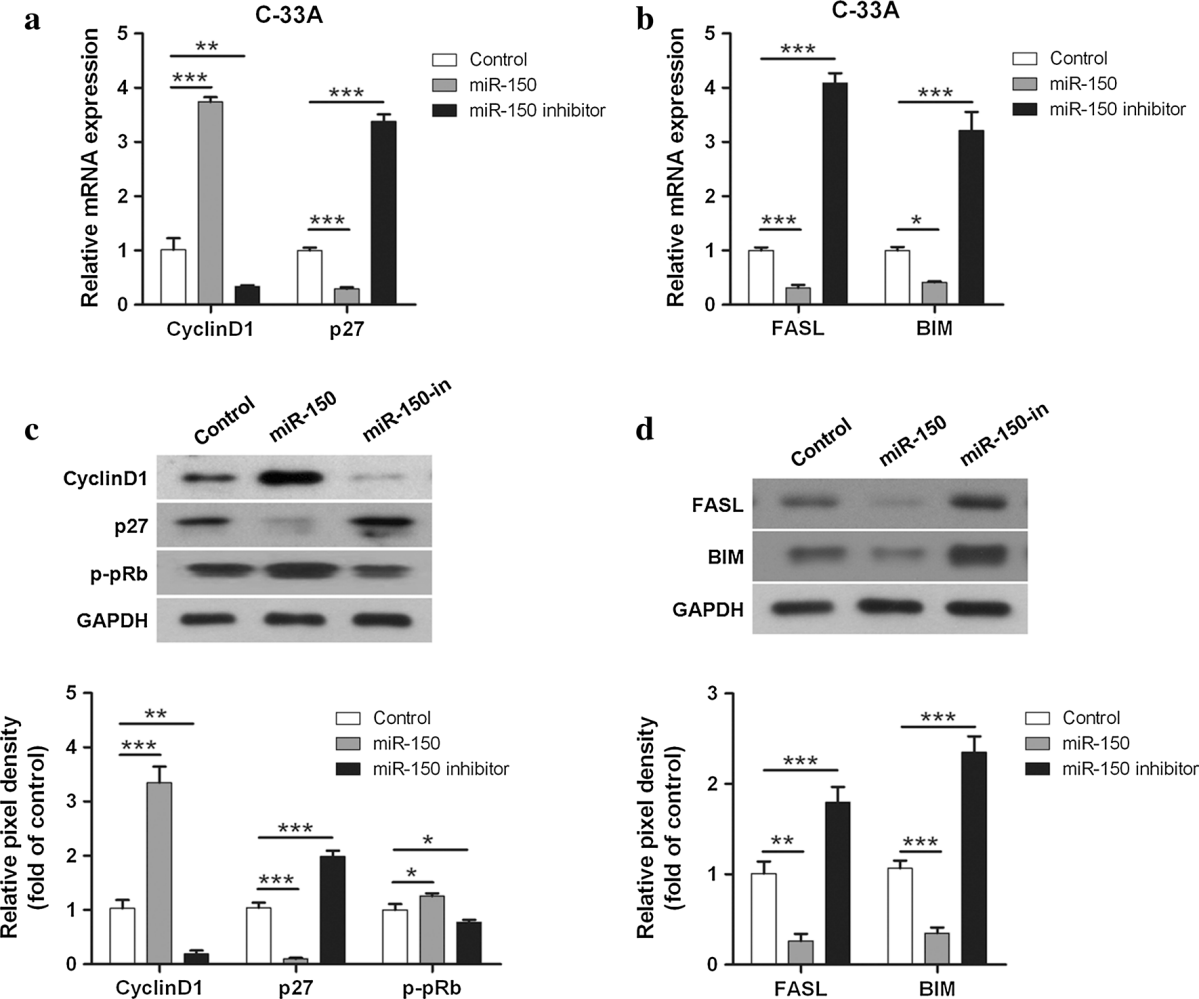
miR-150 affects the expression of proteins involved in cell proliferation and apoptosis. **a** The mRNA expression of cell cycle-related genes CyclinD1 and p27 in the three C-33A sub-cell lines (*control*, *miR-150*, *miR-150 inhibitor*) was measured by RT-PCR. Mean values ± SD for 3 independent experiments is shown. **b** The mRNA expression of apoptosis-related genes FASL and BIM in these three sub-cell lines from 3 independent experiments was evaluated by RT-PCR. Mean values ± SD for 3 independent experiments are shown. **c** The expression of Cyclin D1, p27, GAPDH, and phophorylated pRb (p-pRb) in the sub-cell lines was determined by western blot, and the pixel densities of these proteins were analyzed and presented by histograms (*bottom*). Mean values ± SD for 3 independent experiments is shown. **d** The expression of FASL, BIM, and GAPDH in the sub-cell lines was determined by western blot, and the pixel densities of these proteins were analyzed from 3 independent experiments and presented by histograms (*bottom*)

Since miR-150 promotes the survival and cell cycle progression, expression of several related proteins was determined next in the three sub-cell lines. mRNA expression of two cell cycle-related genes CyclinD1 and p27, as well as two cell apoptosis-related genes FASL and BIM, was measured by qRT-PCR. miR-150 mimics significant upregulated CyclinD1 mRNA expression and reduced the mRNA expression of p27, FASL, and BIM (Fig. 4a, b). In contrast, miR-150 inhibitors decreased the CyclinD1 expression and increased the expression of p27, FASL, and BIM (Fig. 4a, b). Consistent with mRNA results, the protein level of CyclinD1 was increased by miR-150 mimics and reduced by miR-150 inhibitors, whereas protein levels of p27, FASL, and BIM were reduced by miR-150 mimics and increased by miR-150 inhibitors (Fig. 4c, d). Moreover, the phosphorylated pRb, a regulator of cell cycle, was increased by miR-150 and decreased by miR-150 inhibitors (Fig. 4c).

### miR-150 targets FOXO4 in cervical cancer cells

**Fig. 5:**
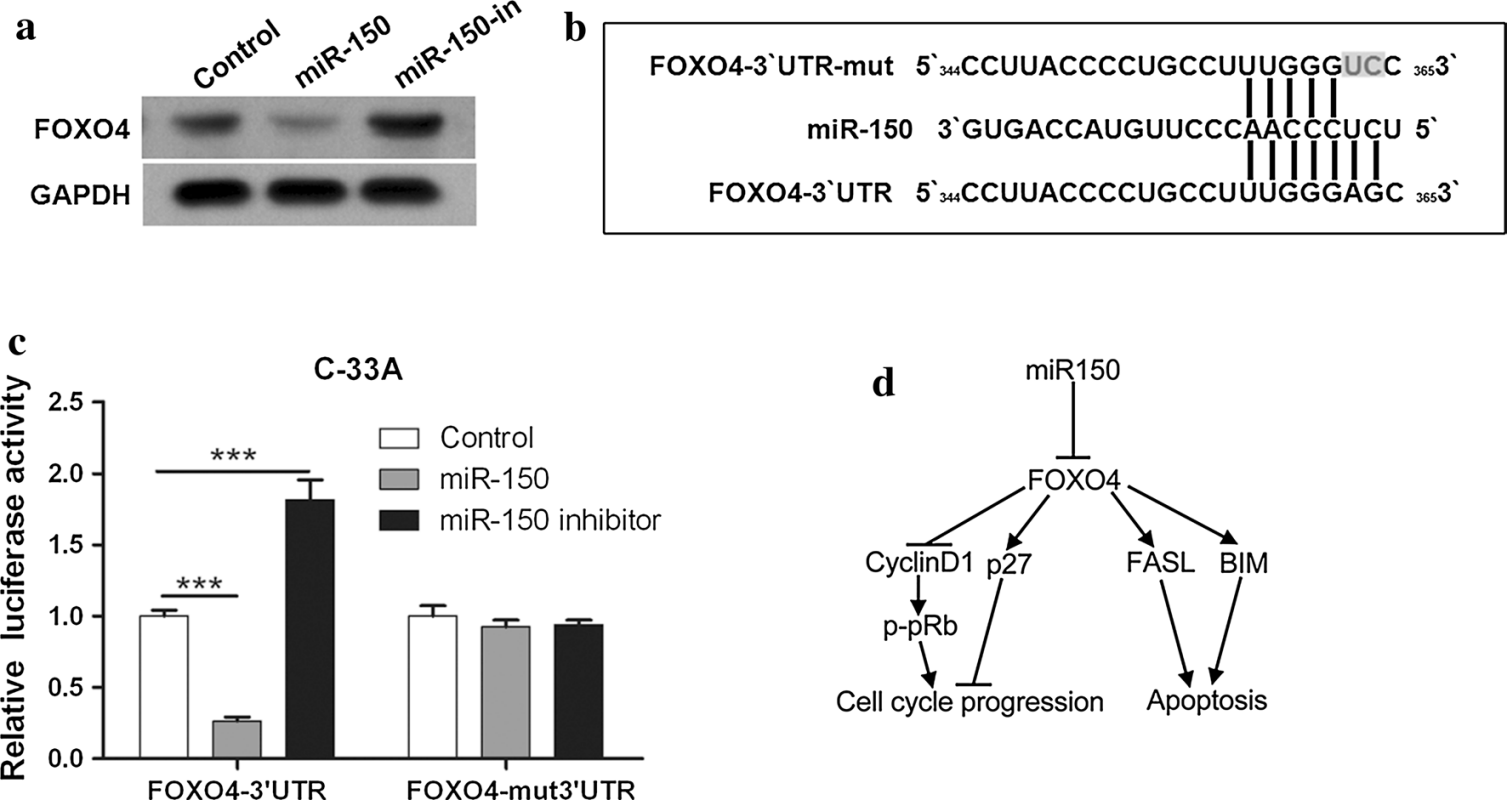
miR-150 directly targets the 3′UTR of FOXO4. **a** The expression of FOXO4 in three C-33A sub-cell lines was determined by western blot. **b** The sequence of the wild type and mutant 3′ UTR of FOXO4 (mut-3′UTR) for luciferase reporter assay are shown. **c** Three C-33A sub-cell lines were transfected with reporter plasmids containing wild type or mutant 3′UTR of FOXO4 for 48 h and the luciferase activity was measured. Mean values ± SD for 3 independent experiments is shown. **d** Hypothetic illustration of how miR-150 promotes cervical cancer cell cycle progression and survival by targeting FOXO4

miRNA plays important roles in cell proliferation, differentiation, apoptosis and other biological processes by targeting the 3′UTR site of multiple genes, therefore regulating their transcription. FOXO4 is a predicted target gene of miR-150 (http://www.microrna.org) and we found significant downregulation of FOXO4 in C-33A cells expressing miR-150 mimics and the upregulation of FOXO4 in the cells expressing miR-150 inhibitors (Fig. 5a). To confirm that miR-150 directly targets the 3′UTR of FOXO4, two luciferase reporter plasmids containing wild type or mutant 3′ UTR were constructed (Fig. 5b). In C-33A cells overexpressing miR-150, the luciferase activity regulated by wild type 3′UTR of FOXO4 was significantly reduced and miR-150 inhibitors increased the luciferase activity (Fig. 5c). Moreover, miR-150 mimics and inhibitors did not affect luciferase activity regulated by mutant 3′UTR of FOXO4 (Fig. 5c), indicating that miR-150 directly targets FOXO4.

## Discussion

Although a variety of altered miRNAs have been identified in cervical carcinomas, their underlying functions and mechanisms in cervical cancer development have not been fully studied. Among 70 dysregulated miRNAs identified in cervical carcinomas, miR-150 is upregulated by above 2.5 times as compared with healthy donors [9]. Our results add further information about the correlation between increased miR-150 and cervical cancer progression. Importantly, we observed pro-cancer effects of miR150 as miR-150 promotes cervical cancer growth and survival. Here, FOXO4 is identified as a direct target of miR-150 which also regulates the expression of CyclinD1, p27, BIM, and FASL, as well as the phosphorylation of pRb. Based on the fact that activated FOXO4 directly or indirectly regulates the expression of CyclinD1, p27, BIM, and FASL [19–24], we propose a hypothesis that miR-150 induces the transcriptional arrest of FOXO4 by binding to 3′-UTR of its mRNA, therefore reduces p27, FASL, BIM and pRb activation, and increases CyclinD1, finally leading to cell cycle progression and survival (Fig. 5d). Our findings highlight the involvement and crucial role of miR-150 in cervical cancer development.

It has been well documented that aberrant expression of miR-150 is associated with cancer development and progression through regulating oncogenes or tumor suppressor genes [13, 25–27]. Different features of miR-150 expression in different solid cancer types have been shown in many studies. Decreased level of miR-150 has been found in pancreatic cancer [28], esophageal cancer [16], colorectal cancer [29–31], and liver cancer [32, 33], while elevated expression of miR-150 has been found in gastric cancer [12, 34], breast cancer [18, 35], and non-small cell lung cancer [14, 17, 36]. Previous studies [9, 10], together with our finding here, confirm that miR-150 is upregulated in cervical cancer. Moreover, our data demonstrated that increased miR-150 expression is also correlated with the processed stages of cervical cancer and revealed a novel role of miR-150 in cervical cancer progression, which involves regulation of cell cycle and apoptosis.

Pro-survival and -growth functions of miR-150 with distinct mechanisms have been demonstrated in breast, gastric and lung cancer cells [12, 14, 18]. Huang et al. has shown that miR-150 overexpression promotes human breast cancer cell growth, clonogenicity, and reduces apoptosis by targeting the pro-apoptotic purinerigic P2X7 receptor and that reduction of miR-150 suppresses tumor growth in xenograft breast tumor mice [18]. Ectopic expression of miR-150 increases the growth of gastric cancer cells and promotes tumorigenesis in vivo [12]. Inhibition of miR-150 in lung cancer cells effectively suppresses cell proliferation and promotes apoptosis [14]. Here, miR-150 overexpression in cervical cancer cells promotes cell growth and survival, while miR-150 inhibition delays cell proliferation and induces more apoptosis. Moreover, miR-150 reduces the mRNA and protein expression of two pro-apoptotic genes BIM and FASL, suggesting that miR-150 promote cell survival through downregulating these genes. Furthermore, miR-150 enhances the cell cycle progression from the G1/G0 to S phase which is related to the decrease of p27 and the increase of CyclinD1.

It has been identified that miR-150 targets the 3′UTR of multiple genes which play important roles in cell proliferation, clonogenicity, invasion, intercellular adhesion, and apoptosis. In gastric cancer cells, miR-150 promotes cell survival by repressing the transcription of EGR2, a pro-apoptotic gene [12]. miR-150 also targets the 3′UTR of p53 and thus regulates its expression, leading to cell survival [14]. 3′UTR of pro-apoptotic purinergic P2X7 is targeted by miR-150 in breast cancer cells [18, 35]. Our work identified FOXO4 as a novel target of miR-150 as miR-150 suppressed the transcriptional process of FOXO4 through targeting the 3′UTR of its mRNA. FOXO4, a member of the class O of forkhead box transcription factors (FOXOs), is an important downstream target of the insulin-phosphatidylinositol 3-kinase (PI3K)–serine–threonine kinase AKT/protein kinase B (AKT/PKB) signaling pathway which can protect cells against apoptosis [19]. Enforced FOXO4 activity induces apoptosis through regulation of various pro-apoptotic genes, such as FASL, BIM, Bcl-6, and PUMA, and modulates cell cycle progression through influencing p27, p130Rb2, CyclinD1, and CyclinG2 [19]. Since miR-150 overexpression modulates the expression of CyclinD1, p27, FASL, and BIM, it is possible that these regulation is caused by miR-150-mediated FOXO4 targeting (Fig. 5d).

## Conclusion

In summary, our results demonstrate the important role of elevated miR-150 in cervical cancer progression and reveal that miR-150 promotes cervical cancer cell growth and survival by targeting FOXO4. Moreover, our data also suggest that miR-150 can be used as a potential therapeutic target which may improve the treatment of cervical cancer.

## Availability of supporting data

The datasets supporting the results of this article are available in the NCBI-GEO repository, GSE30656 [10]. http://www.ncbi.nlm.nih.gov/geo/query/acc.cgi?acc=GSE30656.

## Abbreviations

miR-150: microRNA-150
FOXO: the class O of forkhead box transcription factors
3′ UTR: 3′ untranslated region
EGR2: early growth response factor 2
MUC4: mucins 4
ZEB1: zinc‑finger E‑box binding homeobox 1
P2X7: P2X purinoceptor 7
SRCIN1: SRC kinase signaling inhibitor 1
BAK1: BRI1-associated receptor kinase 1
TUNEL: Terminal deoxynucleotidyl transferase dUTP nick end labeling
PI3K: phosphatidylinositol 3-kinase
AKT/PKB: serine–threonine kinase AKT/protein kinase B
